# Morphological and molecular characterization of some pumpkin (*Cucurbita pepo* L.) genotypes collected from Erzincan province of Turkey

**DOI:** 10.1038/s41598-022-11005-1

**Published:** 2022-04-26

**Authors:** Halil İbrahim Öztürk, Veysel Dönderalp, Hüseyin Bulut, Recep Korkut

**Affiliations:** 1 Vocational School of Health Services, Erzincan Binali Yıldırım University, Erzincan, Turkey; 2 Erzincan Horticultural Research Institute, Erzincan, Turkey

**Keywords:** Plant breeding, Plant genetics

## Abstract

Plant genetic resources constitute the most valuable assets of countries. It is of great importance to determine the genetic variation among these resources and to use the data in breeding studies. To determine the genetic diversity among genotypes of *Cucurbita pepo* L. species of pumpkin, which is widely grown in Erzincan, 29 different pumpkin genotypes collected were examined based on the morphological parameters and molecular characteristics. SSR (Simple Sequence Repeat) markers were used to determine genetic diversity at the molecular level. The analysis of morphological characterization within genotypes showed a wide variability in morphological traits of plant, flower, fruit, and leaf. In the evaluation performed using SSR markers, all primers exhibited polymorphism rate of %100. Seven SSR markers yielded a total of 15 polymorphic bands, the number of alleles per marker ranged from 2 to 3, and the mean number of alleles was 2.14. Polymorphic information content (PIC) ranged from 0.06 (GMT-M61) to 0.247 (GMT-P41), and the mean PIC value per marker was 0.152. Cluster analysis using Nei's genetic distance determined that 29 genotypes were divided into 4 major groups. The present findings have revealed the genetic diversity among pumpkin genotypes collected from Erzincan province and may form the basis for further breeding studies in pumpkin.

## Introduction

The family Cucurbitaceae comprises about 118 genera and 825 species^1^. The genus Cucurbita belonging to this family are among the leading ones that show great diversity in morphological characteristics. This genus consists of 22 wild and 5 cultivated species^2^. *C. maxima* Duch. (winter squash), *C. moschata* Duch. ex Lam. (butternut squash), *C. pepo* L. (pumpkin/summer squash), *C. argyrosperma* Hubersyn. *C. mixta* Pang and *C. ficifolia* Bouche are important cultivars^3^. *Cucurbita pepo* L. is an important species of Cucurbitaceae family with high economic value and genetic diversity^4^ and shows a wide variation in fruit characteristics such as fruit size, shape and color. Although Turkey is outside the area of primary genetic diversity for Cucurbita species, its geographical location and favorable ecological conditions have allowed Cucurbita species with significant genetic diversity over the years^5^. However, despite the agricultural and biological importance of squash/pumpkin (Cucurbita spp.) species, molecular studies have been very limited so far. Today, the widespread use of biotechnological methods has provided many advantages in crop breeding. Different DNA markers have been used successfully in diversity studies evaluating inter- and intra-species genetic relationships. Many studies have been conducted to examine genetic diversity among Cucurbita species using various molecular markers such as Amplified fragment length polymorphism (AFLP)^6^, Random amplification of polymorphic DNA (RAPD)^7^, Inter Simple Sequence Repeat (ISSR)^8^, Sequence related amplified polymorphism (SRAP)^9^, and Simple sequence repeat (SSR)^10^. Allozymes and different DNA marker systems (RFLP, AFLP, ISSR) were used to detemine genetic variability within *Cucurbita pepo* L. species^8,11,12^. Most marker systems used to date have limitations associated with their dominant and/or unreliable nature. Simple sequence repeats (SSRs) are suitable to detect variation within varieties since they are reliable, co-dominant and highly polymorphic as well as detect high levels of allelic diversity^13^. After these markers were first found in humans^14^, they began to be used in other living organisms as well. SSRs are repetitive DNA sequences of 1–6 base pair units^15,16^, with abundance abundant in the genome. Certain SSR markers have functional significance in chromatin organization, regulation of gene activity, and recombination^17^, but they are more often apparently randomly distributed in the nonfunctional genomic regions. SSR markers can be used effectively in population genetics and gene mapping studies because of their advantages as an informative marker system including requiring small amounts of DNA, being codominant and stable, being abundant and scattered throughout the genome, being reproducible and suitable for automation, and having a high level of polymorphism^18^. The SSR technique has successfully been used in the assessment of genetic diversity in cucurbit species such as pumpkin/squash^19–22^, bowler^23^, snake melon^24^ watermelon^25,26^, bitter melon^27^, cucumber^28^. The rate of foreign fertilization in pumpkin is very high. Due to foreign pollination, lines different from the original seed may occur, leading an increased genetic variation. Over time, pumpkin cultivars have spread to the regions of our country with both natural and artificial selections and have been formed from different populations in these regions. This type of plant genetic resources in our country establishes the basis of genetic materials of breeding studies. However, it is important to prevent the disappearance of such local genetic resources to be used in breeding studies. A comprehensive characterization study consisting of morphological and molecular parameters has not yet been carried out in Erzincan province. In this study, it was aimed to determine the degree of genetic relationship at the molecular level by using SSR markers as well as the morphological characteristics of certain pumpkin genotypes grown in Erzincan province.

## Material and method

### Plant material

In this study, the 29 pumpkin genotypes were collected from different regions of Erzincan province (Table 1). Seedlings of 29 different genotypes were produced in the unheated greenhouse of the Erzincan Horticultural Research Institute. Morphological and molecular identification studies of 29 local pumpkin genotypes collected were performed. Experimental research and field studies on plants, including the collection of plant material, complies with relevant institutional, national, and international guidelines and legislation. This study was carried out within the scope of the project. Therefore, all permissions for the collection of plant material and field studies were obtained through the Coordinator of Scientific Research Projects of Erzincan Binali Yıldırım University.

**Table 1:** Coordinate information of the regions where pumpkin genotypes were collected.

Number	Genotype code	Location	Altitude (m)	Latitude (° ′)	Longitude (°′)
1	≠ 1	Bahçeliköy	1371	39°45′	39°20′
2	≠ 2	Bahçeliköy	1371	39°45′	39°20′
3	≠ 3	Bahçeliköy	1371	39°45′	39°21′
4	≠ 4	Bahçeliköy	1371	39°45′	39°21′
5	≠ 6	Çatalarmut	1440	39°48′	39°18′
6	≠ 7	Çatalarmut	1440	39°48′	39°18′
7	≠ 8	Çatalarmut	1440	39°48′	39°18′
8	≠ 9	Çatalarmut	1441	39°48′	39°18′
9	≠ 10	Çatalarmut	1442	39°48′	39°18′
10	≠ 13	Çatalarmut	1443	39°48′	39°18′
11	≠ 14	Çayırlı	1547	39°50′	40°00′
12	≠ 23	Çayırlı	1547	39°50′	40°00′
13	≠ 25	Üzümlü	1290	39°41′	39°41′
14	≠ 26	Üzümlü	1290	39°41′	39°41′
15	≠ 27	Üzümlü	1290	39°41′	39°41′
16	≠ 29	Üzümlü	1290	39°41′	39°41′
17	≠ 30	Üzümlü	1290	39°41′	39°41′
18	≠ 32	Üzümlü	1290	39°41′	39°41′
19	≠ 34	Üzümlü	1290	39°41′	39°41′
20	≠ 36	Üzümlü	1290	39°41′	39°41′
21	≠ 38	Cevizli	1400	39°43′	39°21′
22	≠ 40	Cevizli	1400	39°43′	39°21′
23	≠ 41	Cevizli	1400	39°43′	39°21′
24	≠ 42	Cevizli	1400	39°43′	39°21′
25	≠ 46	Cevizli	1400	39°43′	39°21′
26	≠ 49	Ortayurt	1262	39°61′	39°58′
27	≠ 50	Ortayurt	1263	39°61′	39°58′
28	≠ 51	Ortayurt	1264	39°61′	39°58′
29	≠ 53	Ortayurt	1265	39°61′	39°58′

### Determination of morphological properties

Morphological identification studies were carried out in the fields and laboratories of the Erzincan Horticultural Research Institute. Genotypes were evaluated in terms of different phenotypic characteristics including plant (growth habit, branching, degree of branching), leaf (leaf blade: size, incisions, density of green color of upper surface, marbling, mottling), petiole (attitude of petiole, green color, length, thickness, degree of prickles) and fruit (shape, major color, intensity of major color, number of colors, diameter, length, indices) traits.

### SSR analysis

For SSR analysis, plant genomic DNA was isolated with minor modifications to the protocol defined by Saghai-Maroof^29^. 50 ml isolation buffer was prepared and heated to 70 °C in a water bath and 100 μl of β-mercaptoethanol [Merck®] was added into it. The samples were weighed on a precision balance to 0.3 g and grinded with liquid nitrogen. The ground samples were taken into 2.0 ml eppendorf tubes, 1000 μl of isolation buffer solution was added, and incubated in a 70 °C water bath for 60 min by turning upside down every 10 min. 750 μl of chloroform: isoamyl alcohol (24:1) was added to the samples and slightly turned upside down. Mixed samples were centrifuged at 14,000 rpm for 20 min at 4 °C. At the top layer (supernatant) of the three layers formed was removed using a pipette and transferred to new eppendorf tubes. The same proportion of chloroform:isoamyl alcohol was added again to the supernatant and centrifuged at 14,000 rpm for 20 min at 4 °C. The upper phase was transferred to new eppendorf tubes and 100 μl of 10 M ammonium acetate and 100 μl of 3 M sodium acetate were added. 2.5 times of isopropanol (− 20 °C) was added to the resulting mixture and slightly turned upside down. When the DNA pellet was seen, the eppendorf tubes were centrifuged at 14,000 rpm for 20 min at 4 °C. The supernatant was obtained by removing the liquid part from the tubes. The tubes were centrifuged at 14,000 rpm for 1 min at 4 °C and then left to dry in the incubator at 37 °C for 15 min. 100 µl of TE buffer was added to the genomic DNAs obtained from the samples and stored at + 4 °C. To measure the purity of DNA samples, 4 µl of DNA + 996 µl of TE buffer was added, and absorbance (A) values were read in the spectrophotometer at 260 nm and 280 nm wavelengths. DNA samples with a 260/280 value between 1.1 and 1.8 were labeled as pure DNA. Using the formula 50 (multiplication coefficient for DNA) × 250 (dilution coefficient) × OD 260 (read value at 260 nm), the amount of DNA in the stock was calculated and working solutions containing 50 ng/l DNA were prepared from the stock DNA. Information about the SSR primers used in our study is given in Table 2.

**Table 2:** Information on SSR primers.

Primers	Repeat motif	Forward primer (3′–5′) Reverse primer (5′–3′)
CMTp18	(TC)_17_	F: ACACCTTCGCTTCCGACATC R: TGACATCACTCCGGCAACTC
CMTm25	(TTCTTCT)_5_	F: CTGACGTCGCTACTCATAGCA R: TGAAGCTTTCAGAAATGAATGTG
CMTm30	(AAG)_5_ + (CAC)_7_	F: CAAACCATAACTTCCAG R: AGGTCCATATTTGACG
CMTp41	(GCC)_8_ + (CCT)_4_	F: GGAGGCCTTGGAATGATAGG R: TTCTCTCAACCACCGTCACC
CMTm61	(GGA)_4_ + (AAAA)_4_	F: GCCATTATTCCACTCCATGC R: TGCCTGCACCTGTTTTAGC
CMTp68	(TC)_10_ + (GGCTTC)_6_	F: ATTGATTGGGACGTGAGGAA R: CACACCCATTTCATTTTGACC
CMTm259	(AG)_8_	F: ACCTCGAGGAAGCAAAAATG R: ATGGAGACGCGCAAGTAGAT

### Data analysis

The PIC values of each SSR markers were calculated using the formulas given below. Allelic data were used to compute PIC value of SSRs, the codominant molecular marker system, using the Power Marker^30^ program^31^. Genetic variation within genotypes was determined by Nei's gene diversity index^32^, Shannon information index^33^, and the Popgen program^34^. NTSYS-pc version 2.11 f^35^ was used for the clustering analysis of the data set obtained from the SSR markers. The clustering was performed with the SAHN subprogram using the unweighted pair group method with arithmetic Mean (UPGMA) method. The STRUCTURE 2.2 program was used to determine the genetic structures of the genotypes^36^. In many genetic diversity studies with pumpkin, genotypes are successfully separated into groups using the STRUCTURE program^37,38^. The F-statistic (FST) value reflects the variation between sub-populations^39^. By using the GenAlex program, principal coordinate analysis was performed to better understand the diversity among genotypes.

## Results

### Morphological properties of pumpkin genotypes

In this study, 29 pumpkin genotypes belonging to *Cucurbita pepo* were collected from different locations in Erzincan province. This pumpkin population has been characterized according to morphological and molecular traits. Since changes in morphological traits occurred in response to external conditions, it is important to support these morphological variations with molecular studies. Morphological features of genotypes are given in Tables 3, 4 and 5. It was observed that there were significant morphological differences in plant phenotype, leaf, flower and fruit characteristics among the collected *Cucurbita pepo* genotypes. The plant growth habit was considered as creeping in 14 genotypes, semi-creeping in 10 genotypes and shrub in 5 genotypes. Branching was determined in 24 genotypes, while other 5 genotypes did not have branching characteristics. Leaf attitude of petiole was identified as erect in 16 genotypes and semi-erect in 13 genotypes. In addition, pumpkin genotypes showed high variation in terms of leaf characteristics such as leaf blade size, incisions of leaf blade, green color of leaf blade and green color of petiole. Incisions of leaf blade was weak in 11 genotypes, medium in 9 genotypes, strong in 1 genotype and very strong in 1 genotype, whereas in 7 genotypes incisions of leaf blade were absent (Table 3). As with other morphological features, it was observed that there was variation among genotypes in terms of flowers (male and female). It was determined that approximately 10 of the genotypes had ring at inner side of corolla and that there were no rings in the female flowers of 19 genotypes. In terms of pistil color in female flowers, genotypes are divided into 2 groups as yellow and orange. It was observed that in vast majority (approximately 76%) of the genotypes pistil colour was yellow. Based on the expression of colored ring at inner side of corolla of male flowers, genotypes are divided into 5 groups as absent, weak, medium, strong and very strong. It was observed that the majority of the genotypes (11 genotypes) had strong expression of colored ring at inner side of corolla. Genotypes were divided into 3 groups as yellow, yellow-green and green according to color of pedicel of male flower. It was determined that 12 genotypes had yellow, 9 genotypes had yellow-green and 8 genotypes had green color. Differences were determined between genotypes according to the hairiness of pedicel of male flower. Genotypes were divided into 3 groups based on this trait. 9 genotypes were classified as weak, 11 genotypes as medium and 9 genotypes as strong (Table 4). In addition, pumpkin genotypes showed high variation in fruit shapes and skin colours. It was determined that fruit shape of 8 genotypes were transverse elliptical, 8 genotypes were wide elliptical, 6 genotypes were elliptical, 4 genotypes were transverse wide elliptical, 2 genotypes were cylindrical and 1 genotype was ovoid. Four different colors were determined as the major colour of skins of the pumpkin genotypes: cream (6 genotypes), yellow (2 genotypes), orange (1 genotype) and green (20 genotypes) (Table 5).

**Table 3:** Plant and leaf morphological parameters of pumpkin genotypes.

Genotypes	Plant			Leaf			
	Growth habit	Branching	Degree of branching	Position of the leafstalk	Leaf blade size	Incisions	Intensity of green color
≠ 1	Trailing	Present	Medium	Semi vertical	Small	Absent	Dark
≠ 2	Trailing	Present	Medium	Vertical	Medium	Medium	Medium
≠ 3	Bushy	Absent	Weak	Semi vertical	Medium	Medium	Dark
≠ 4	Semi trailing	Present	Weak	Semi vertical	Small	Absent	Medium
≠ 6	Semi trailing	Present	Weak	Vertical	Small	Medium	Dark
≠ 7	Semi trailing	Present	Weak	Semi vertical	Small	Strong	Dark
≠ 8	Trailing	Present	Medium	Semi vertical	Small	Medium	Dark
≠ 9	Semi trailing	Present	Weak	Vertical	Medium	Shallow	Dark
≠ 10	Trailing	Present	Weak	Vertical	Small	Shallow	Medium
≠ 13	Semi trailing	Present	Weak	Semi vertical	Small	Shallow	Dark
≠ 14	Semi trailing	Present	Weak	Semi vertical	Small	Medium	Dark
≠ 23	Trailing	Present	Medium	Vertical	Small	Shallow	Dark
≠ 25	Trailing	Present	Strong	Semi vertical	Small	Absent	Dark
≠ 26	Bushy	Absent	Weak	Semi vertical	Medium	Shallow	Dark
≠ 27	Trailing	Present	Medium	Vertical	Small	Shallow	Medium
≠ 29	Trailing	Present	Weak	Vertical	Small	Shallow	Medium
≠ 30	Trailing	Present	Medium	Vertical	Small	Absent	Medium
≠ 32	Trailing	Present	Medium	Vertical	Small	Shallow	Medium
≠ 34	Trailing	Present	Weak	Semi vertical	Small	Shallow	Dark
≠ 36	Semi trailing	Present	Weak	Vertical	Small	Shallow	Dark
≠ 38	Semi trailing	Present	Weak	Vertical	Small	Medium	Dark
≠ 40	Trailing	Present	Strong	Semi vertical	Small	Absent	Dark
≠ 41	Trailing	Present	Strong	Vertical	Small	Absent	Dark
≠ 42	Semi trailing	Present	Weak	Vertical	Small	Medium	Dark
≠ 46	Bushy	Absent	Weak	Vertical	Small	Absent	Medium
≠ 49	Bushy	Absent	Weak	Vertical	Small	Medium	Dark
≠ 50	Trailing	Present	Medium	Semi vertical	Small	Medium	Medium
≠ 51	Semi trailing	Present	Weak	Vertical	Small	Shallow	Medium
≠ 53	Bushy	Absent	Weak	Semi vertical	Small	Very strong	Dark

**Table 4:** Flower morphological parameters of pumpkin genotypes.

Genotypes	Female flower		Male flower			
	Petal inner circle	Pistil color	Petal inner circle color grade	Inner circle color	The length of the flower stalk	Hairiness on the flower stalk
≠ 1	Absent	Yellow	Strong	Yellow-green	Short	Weak
≠ 2	Absent	Yellow	Medium	Yellow	Medium	Strong
≠ 3	Absent	Orange	Strong	Yellow	Medium	Medium
≠ 4	Absent	Yellow	Strong	Yellow	Medium	Medium
≠ 6	Absent	Yellow	Strong	Yellow	Short	Medium
≠ 7	Present	Yellow	Strong	Yellow	Medium	Weak
≠ 8	Absent	Yellow	Strong	Yellow	Medium	Medium
≠ 9	Present	Yellow	Strong	Yellow	Medium	Medium
≠ 10	Present	Yellow	Medium	Yellow	Short	Medium
≠ 13	Absent	Yellow	Medium	Yellow	Medium	Strong
≠ 14	Present	Yellow	Medium	Yellow-green	Medium	Strong
≠ 23	Absent	Orange	Absent	Yellow	Long	Medium
≠ 25	Present	Orange	Slight	Yellow-green	Long	Medium
≠ 26	Present	Orange	Strong	Green	Long	Strong
≠ 27	Present	Orange	Medium	Yellow-green	Long	Weak
≠ 29	Absent	Yellow	Very strong	Green	Medium	Strong
≠ 30	Absent	Yellow	Slight	Yellow	Medium	Weak
≠ 32	Absent	Orange	Slight	Yellow-green	Medium	Strong
≠ 34	Absent	Yellow	Very strong	Green	Medium	Weak
≠ 36	Absent	Yellow	Medium	Yellow	Medium	Strong
≠ 38	Present	Yellow	Medium	Yellow-green	Medium	Strong
≠ 40	Absent	Yellow	Medium	Yellow-green	Medium	Medium
≠ 41	Absent	Yellow	Strong	Green	Medium	Medium
≠ 42	Present	Yellow	Very strong	Green	Medium	Weak
≠ 46	Absent	Yellow	Absent	Green	Medium	Weak
≠ 49	Absent	Yellow	Absent	Yellow-green	Medium	Weak
≠ 50	Absent	Yellow	Strong	Yellow-green	Medium	Strong
≠ 51	Absent	Yellow	Slight	Green	Medium	Medium
≠ 53	Present	Orange	Strong	Green	Short	Weak

**Table 5:** Fruit morphological parameters of ornamental pumpkin genotypes.

Genotype	Fruit						
	Shape	Main color of skin	Intensity of skin main color	Number of skin color	Diameter	Length	Index
≠ 1	Elliptical	Green	Dark	Two	Large	Long	High
≠ 2	Transverse elliptical	Green	Dark	Three	Large	Long	High
≠ 3	Cylindrical	Orange	Dark	Two	Large	Long	Medium
≠ 4	Wide elliptical	Green	Medium	Three	Large	Long	Medium
≠ 6	Transverse elliptical	Green	Medium	Two	Large	Long	Medium
≠ 7	Transverse elliptical	Green	Medium	Two	Large	Medium	Low
≠ 8	Transverse wide elliptical	Green	Medium	Two	Large	Long	Low
≠ 9	Elliptical	Yellow	Medium	One	Medium	Long	Medium
≠ 10	Wide elliptical	Green	Medium	Two	Large	Long	Medium
≠ 13	Transverse wide elliptical	Green	Medium	Two	Large	Long	Medium
≠ 14	Elliptical	Green	Medium	Three	Medium	Long	High
≠ 23	Transverse wide elliptical	Green	Light	One	Large	Medium	Low
≠ 25	Transverse elliptical	Green	Medium	One	Large	Long	Medium
≠ 26	Elliptical	Cream	Medium	One	Medium	Medium	Medium
≠ 27	Transverse elliptical	Green	Medium	Two	Large	Long	Medium
≠ 29	Wide elliptical	Green	Medium	Two	Large	Long	Low
≠ 30	Transverse wide elliptical	Green	Dark	One	Large	Medium	Low
≠ 32	Transverse elliptical	Green	Medium	Two	Large	Long	Medium
≠ 34	Elliptical	Cream	Medium	One	Medium	Long	Medium
≠ 36	Wide elliptical	Green	Medium	Two	Large	Long	Low
≠ 38	Wide elliptical	Cream	Medium	One	Large	Long	Medium
≠ 40	Wide elliptical	Cream	Light	One	Medium	Long	High
≠ 41	Wide elliptical	Green	Light	One	Medium	Long	Medium
≠ 42	Wide elliptical	Green	Medium	Two	Large	Long	Medium
≠ 46	Ovoidal	Yellow	Medium	One	Medium	Medium	Medium
≠ 49	Elliptical	Cream	Medium	One	Medium	Long	High
≠ 50	Transverse elliptical	Green	Dark	One	Large	Long	Medium
≠ 51	Transverse elliptical	Green	Medium	Two	Large	Long	Low
≠ 53	Cylindrical	Cream	Light	One	Medium	Long	Very high

### SSR analysis

The 7 SSR markers used in our study produced a total of 15 polymorphic bands, the number of alleles per marker ranged from 2 (GMT-P41, GMT-M61, GMT-M259, GMT-P18, GMT-P25 and GMT-M30 markers) to 3 (GMT-P68 marker) and the mean number of alleles was f 2.14 (Table 6). The PIC value ranges from 0.06 (GMT-M61) to 0.247 (GMT-P41), with a mean of 0.152. The markers GMT-P41, GMT-P25 and GMT-P68 were found to be the best among the markers used to discriminate between genotypes due to their higher PIC values. (Table 6).

**Table 6:** Allele number, polymorphic allele number, polymorphism percentage and PIC values of iBPS markers.

Number	Primer	Number of alleles	Major allele frequency	Gene diversity	PIC
1	GMT-P41	2	0.724	0.314	0.247
2	GMT-M61	2	0.966	0.064	0.060
3	GMT-P68	2	0.851	0.212	0.173
4	GMT-M259	3	0.931	0.119	0.105
5	GMT-P18	2	0.879	0.183	0.150
6	GMT-P25	2	0.828	0.283	0.242
7	GMT-M30	2	0.948	0.093	0.084
Mean	2.14	0.875	0.181	0.152	
Total	15				

*PIC* Polymorphic information content.

### Cluster analyzes and principal component analyzes for SSR markers

**Figure 1:**
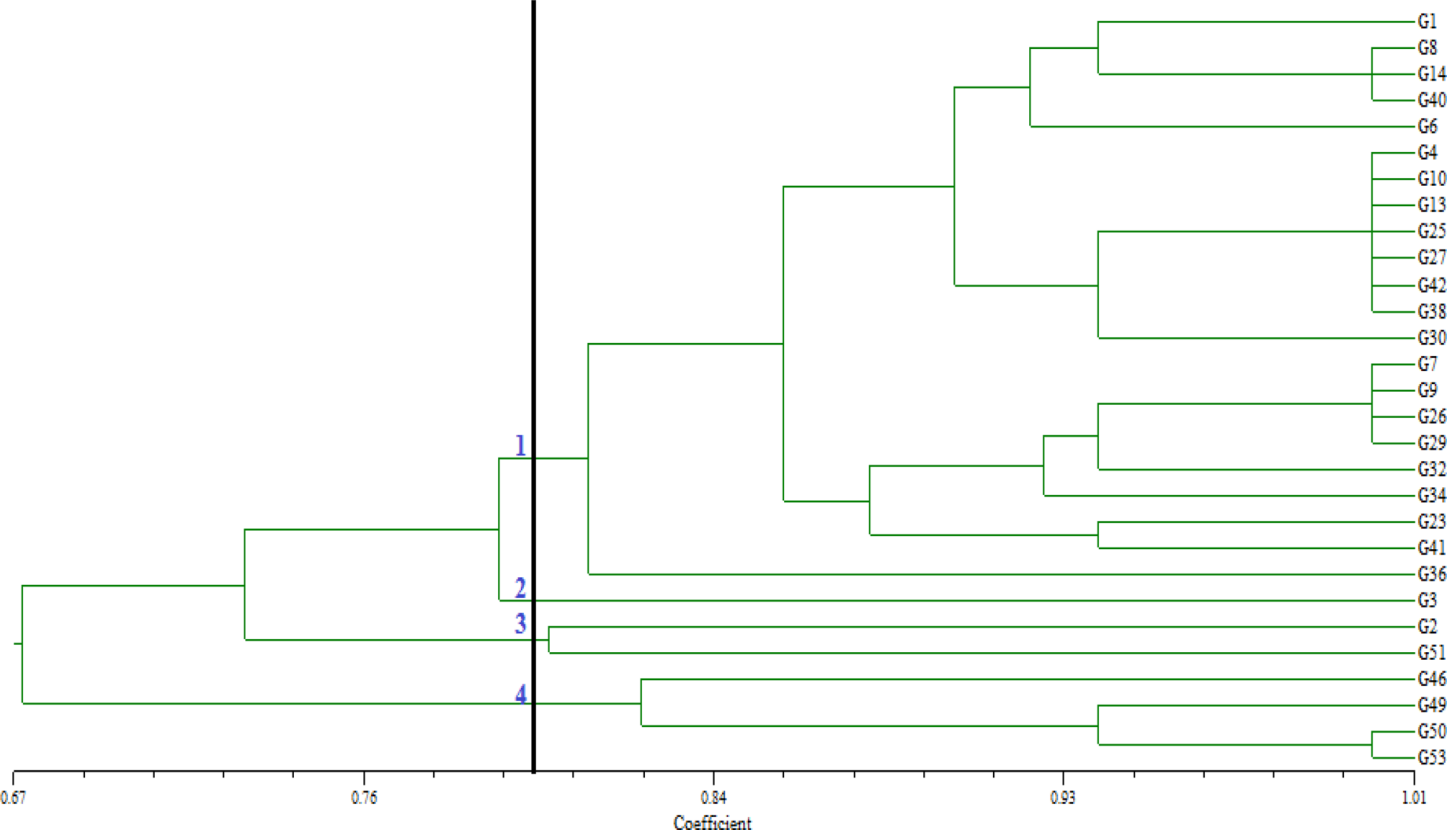
Dendrogram generated by UPGMA method using SSR marker.

Comparative analysis of molecular sequence data enables the determination of proximity or distance between genotypes as well as the construction of a phylogenetic tree for clustering genotypes. For this purpose, cluster analysis was performed between pumpkin genotypes using UPGMA based on Nei's genetic distance. According to the results of this analysis, four major clusters were formed. Dice genetic similarity coefficient was used to estimate genetic diversity. This coefficient is often used to estimate genetic distance. The highest genetic difference (0.63) was found between genotypes ≠ 36 and ≠ 46 genotypes. As a result of the analysis, pumpkin genotypes were divided into four major groups. In the first cluster, mostly genotypes of Bahçeliköy (60%), Cevizli (90%), Çatalarmut (100%), Çayırlı (100%), Üzümlü (100%) and Ortayurt (50%) locations were included. In the second group, only single genotype of Bahçelikoy location (≠ 3) was determined. In the third group, single genotype was found for each of Bahçeliköy (≠ 2) and Ortayurt (≠ 51) locations. In the fourth group, there were 4 genotypes collected from Cevizli (≠ 46) and Ortayurt (≠ 49, ≠ 50 and ≠ 53) locations (Fig. 1).

**Figure 2:**
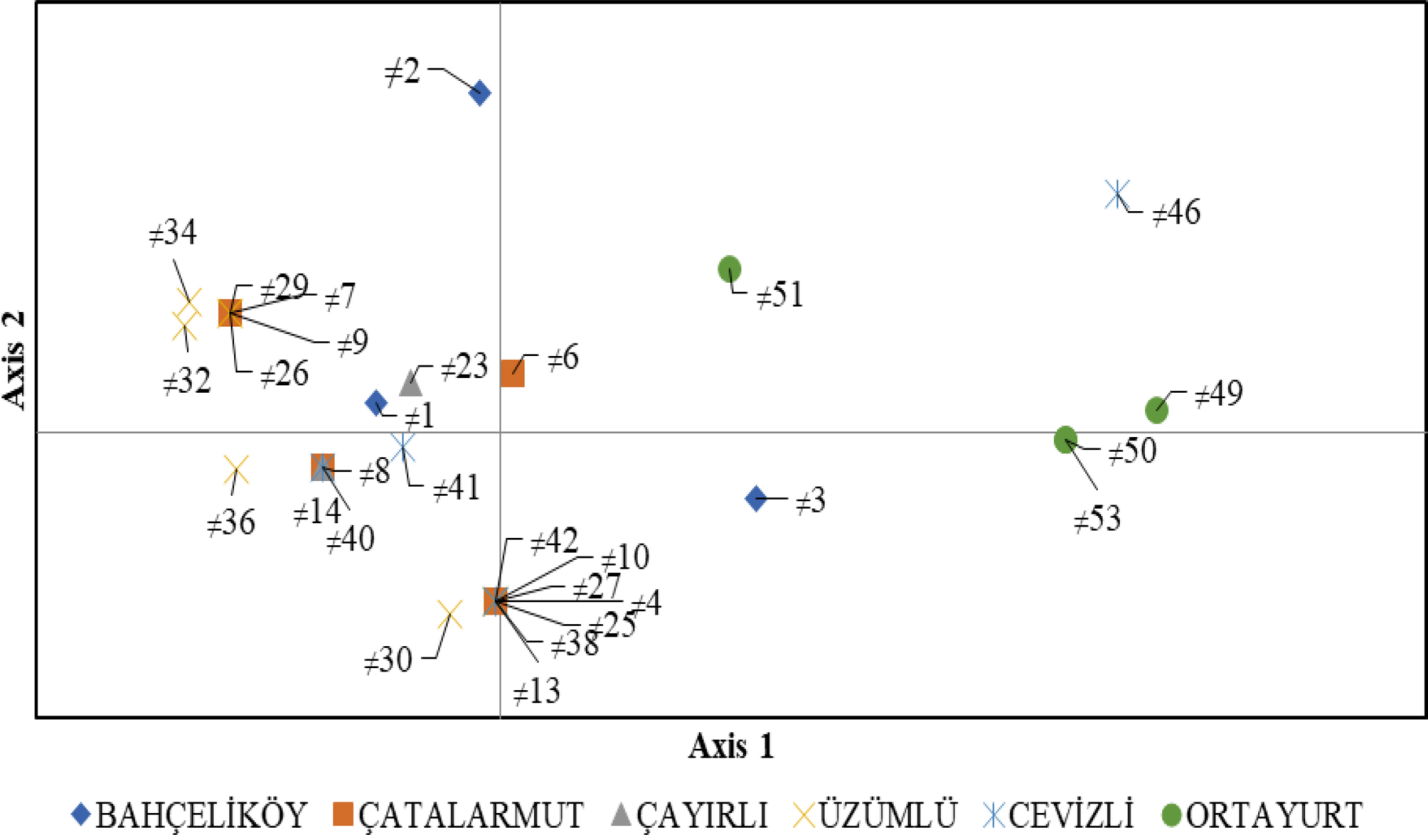
PCA created using the SSR marker and separated on 2-dimensional diagram.

According to present findings, the genotypes Bahçeliköy (≠ 1, ≠ 2), Çatalarmut (≠ 7, ≠ 9), Çayırlı (≠ 23), Üzümlü (≠ 26, ≠ 29, ≠ 32, ≠ 34) were placed on upper left section of the Principle Axis-1. The genotypes Bahçeliköy (≠ 4), Çatalarmut (≠ 8, ≠ 10, ≠ 13), Çayırlı (≠ 14), Üzümlü (≠ 25, ≠ 27, ≠ 30, ≠ 36) and Cevizli (≠ 38, ≠ 40, ≠ 41 ≠ 42) were gathered on lower left section of Axis-1. The genotypes Bahçeliköy (≠ 3) and Ortayurt (≠ 50, ≠ 53) were placed on lower right section of Axis -1. The genotypes Çatalarmut (≠ 6), Cevizli (≠ 46) and Ortayurt (≠ 49, ≠ 51) were gathered on upper right section of Axis-1 (Fig. 2).

### Genetic structure analysis of SSR markers

**Figure 3:**
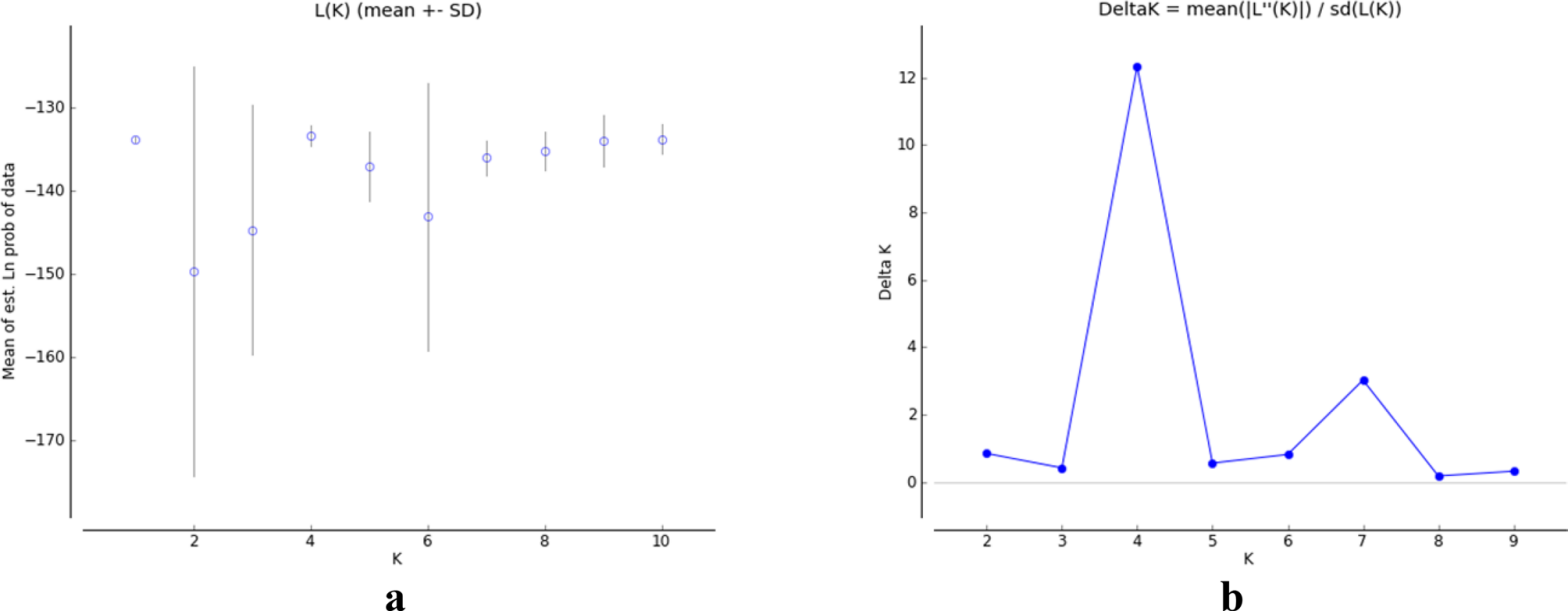
Line plots from the mix model of Ln P(D) and ∆K structure for squash populations (**a**) The average value of the Ln P(D) statistic produced by the structure at each value of K, (**b**) DK.

**Figure 4:**
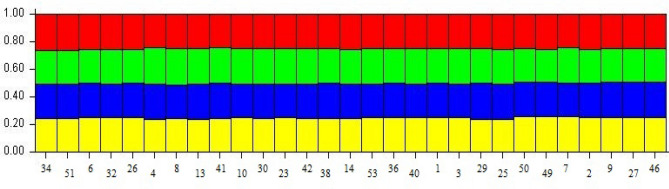
Genetic structure of genotypes according to SSR data (*Cucurbita pepo*) genotypes given in K = 4 are presented in Table 4).

ΔK is used to determine optimal values of K. The highest value in our study was obtained as K = 4 (Fig. 3). The low population size (K value) in our study is thought to be due to the high gene flow between the sample collection regions. Similar results have been reported for the population structure of pumpkin genotypes in other studies^21^. In our study, 22 genotypes were found in the first subpopulation, 1 genotype in the second subpopulation, 2 genotypes in the third subpopulation, and 4 genotypes in the fourth subpopulation (Fig. 4; Table 7). The FST (F-statistics) values in the first, second, third and fourth subpopulations were determined as 0.0399, 0.0217, 0.072 and 0.000, respectively (Table 8).

**Table 7:** Membership coefficient of squash genotypes in four subpopulations.

Genotype	Sub-population			
	I	II	III	IV
≠ 1	0.253	0.249	0.249	0.248
≠ 2	0.250	0.247	0.252	0.251
≠ 3	0.249	0.252	0.251	0.248
≠ 4	0.246	0.251	0.253	0.250
≠ 6	0.251	0.252	0.250	0.247
≠ 7	0.252	0.247	0.251	0.250
≠ 8	0.251	0.253	0.249	0.246
≠ 9	0.251	0.252	0.250	0.248
≠ 10	0.253	0.253	0.248	0.245
≠ 13	0.252	0.251	0.250	0.247
≠ 14	0.250	0.251	0.247	0.251
≠ 23	0.248	0.249	0.249	0.253
≠ 25	0.249	0.249	0.252	0.250
≠ 26	0.251	0.247	0.248	0.254
≠ 27	0.249	0.247	0.253	0.250
≠ 29	0.249	0.251	0.247	0.253
≠ 30	0.254	0.248	0.247	0.251
≠ 32	0.253	0.252	0.248	0.247
≠ 34	0.247	0.246	0.255	0.252
≠ 36	0.253	0.250	0.250	0.248
≠ 38	0.249	0.251	0.252	0.249
≠ 40	0.248	0.251	0.249	0.253
≠ 41	0.249	0.252	0.249	0.250
≠ 42	0.248	0.253	0.245	0.254
≠ 46	0.251	0.244	0.254	0.251
≠ 49	0.244	0.245	0.256	0.255
≠ 50	0.252	0.245	0.251	0.251
≠ 51	0.248	0.246	0.253	0.254
≠ 53	0.250	0.248	0.252	0.250

**Table 8:** Expected heterozygosity and FST values in four squash subpopulations.

Sub-population (K)	Expected heterozygosity	F_ST_
1	0.1917	0.0399
2	0.1928	0.0217
3	0.1953	0.0072
4	0.1953	0.0000
Mean	0.1938	0.0172

## Discussion

Examination of morphological characterization within genotypes showed a wide variation of genotypes in terms of morphological characteristics (plant, flower, fruit, leaf). In many studies of Cucurbitaceae family, it has been emphasized that diversity is high in terms of morphologic characteristics^40–43^. In a similar study by^8^, it has been determined that pumpkin genotypes showed high diversity in terms of fruit characteristics^44^ have showed that major color of the skin was yellow in 21 (24%) pumpkin genotypes green in 2 (2%), green-yellow grayish in 15 (18%), dark yellow -green grayish in 22 (27%), light yellow in 17 (21%) and dark yellow in 4 (5%). It was observed that 7 SSR markers used in pumpkin genotypes yielded a total of 15 bands and the number of alleles per locus was 2.14. The SSR method has been successfully applied to various species to identify genetic relationships^21,45–48^. These markers have proven to effectively improve genetic diversity analysis and are very effective tools in genetic diversity and association studies due to their high polymorphic nature and transferability^49–51^. In similar studies of *Cucurbita pepo* species, researchers have found the mean number of alleles amplified per SSR marker primers as 3^21,52^. The results are similar to the results in our study. In many studies using SSR markers, it has been stated that SSR markers are successful to detect polymorphism and diversity in species belonging to the genus Cucurbita^11,52,53^. Polymorphic information content (PIC) is an important value that evaluates the efficiency of polymorphic loci and determines the discrimination ability of markers. In some studies, the PIC value changed according to the number of SSR markers used and the number of genotype and analysis method. In other studies, with SSR markers, the PIC value was found between 0.49 and 0.75 for melon and between 0.18 and 0.64 for cucumber. Of the markers, PKCT111 was considered the most informative as it showed the greatest genetic variation^54^. In a study conducted in Kenya with 96 pumpkin samples using SSR markers, the mean PIC value was determined as 0.49, and cluster analysis showed that the level of similarity between genotypes was high^55^. Based on genetic structure analysis and UPGMA analysis, 4 groups were identified. Principle component analysis (PCA) presents spatial distribution of relative genetic distance between the populations^56^. In present study, PCA analysis was performed for better and more detailed visualization of the variation within and between the populations. With the aid this method, a 2-D diagram is generated based on closeness or distance matrix between the genotypes and the distances between the resultant groups put forth the actual distances^57^. Expanding our knowledge about genetic variation of genotypes is crucial for crossbreeding studies used to obtain lines resistant to various stress conditions or more productive varieties. Therefore, the assessment of genetic variability in the gene source is the first step, called pre-breeding, to improve and develop superior varieties. SSRs with high polymorphism information content successfully assisted in the differentiation of genotypes in this study. The results of this study suggest that SSR analysis can be used successfully in the estimation of genetic diversity among pumpkin genotypes and potentially be included in future studies examining diversity in a larger collection of pumpkin genotypes from various regions. It is thought that the results of this study will contribute to the existing pumpkin cultivation and conservation of genetic resources in Turkey. The outcomes obtained in this study provide significant findings for the future in marker selection, characterization of genetic source, cultivation and selection of pumpkin genetic source.
